# The development of xenograft glioblastoma implants in nude mice brain

**Published:** 2008-08-15

**Authors:** FM Behar, AV Ciurea, M Chivu, O Zarnescu, R Radulescu, D Dragu

**Affiliations:** * Emergency Clinical Hospital ‘Bagdasar–Arseni’, BucharestRomania; ** National Institute of Virusology ‘Stefan S. Nicolau’, BucharestRomania; *** University of Bucharest, Department of HistologyRomania

**Keywords:** glioblastoma multiformis, xenograft model, U 87 line, athymic mice, stereotactic inoculation

## Abstract

The inefficacity of the actual therapies for *glioblastoma multiformis* stimulates the researchers to search for new and innovative therapies. Therefore, the development of in vivo model for glioblastoma is an essential step during these researches, being a link between cells cultures studies and the first phases of clinical trials.

In this paper, we present several procedures which have been performed for the first time in our country, such as: the cultivation and manipulation of U87MG line, the manipulation of athymic – knock–out mice (NUDE Crl: CD–1 Foxn1), the stereotactic inoculation of glioblastoma cells and finally the development of glioblastoma xenograft in the brain of inoculated nude mice.

These results, which offer to the researchers from our country an in vivo model for glioblastoma, could be the start point for several projects oriented to the development of new therapies in glioblastoma, and could raise the performance of our scientific research to the European level.

## Introduction

*Glioblastoma multiformis* is the most malignant primary brain tumor in adult. The life expectation for a patient with glioblastoma does not usually exceed one year. In spite of the complex treatment applied (neurosurgical resection, radiotherapy, chemotherapy), the outcome is extremely poor, with 100% mortality. The inefficiency of actual therapies stimulates the researchers toward the direction of discovering new innovative therapies. Therefore, the development of in vivo model for glioblastoma represents an essential step during these researches, being a link between the cells cultures studies and the first phases of clinical trials.

In this paper the authors present for the first time in Romania, the development of glioblastoma xenograft in nude mice. Several materials and procedures have been acquired or performed for the first time in our country, such as: the cultivation and manipulation of U87MG line (an international glioblastoma line brought for the first time in Bucharest), the breeding and manipulation of athymic –knock–out mice (NUDE Crl: CD–1 Foxn1, acquired from Charles River laboratories), the stereotactic inoculation of glioblastoma cells and finally the development of glioblastoma in the brain of nude mice, proved by histopathological studies and immunohistochemistry images.

This achievement is the result of the collaboration between the research department in neuroscience of the Clinical Hospital ‘Bagdasar–Arseni’, the National Institute of Virology ‘Stefan S. Nicolau’ and Department of Histology of University of Bucharest. The experiments have been supported by the national research grant CEEX–VIASAN, no.176/2006.

## Material

### Glioblastoma line

The glioblastoma line, U87MG, has been acquired from the European Collection of Cell Cultures (ECACC). This line has been cultivated and frozen in several research centers in Bucharest: Department of basic research in neuroscience from Clinical Hospital ‘Bagdasar–Arseni’, National Institute of Virusology ‘Stefan S. Nicolau’ and National Institute of Biological Sciences.

This line has the following characteristics (according to ECACC description):

- Cell Line Name: U87MG
- Cell Line Description: derived from a malignant glioma from a female patient by explant technique. It is reported to produce a malignant tumour consistent with glioblastoma in nude mice.
- Species: Human
- Tissue: Brain
- Morphology: Epithelial–like
- Sub Culture Routine: Split sub–confluent cultures (70–80%) 1:3 to 1:6 i.e. seeding at 2–4x10,000 cells/cm^2^ using 0.25% trypsin or trypsin/EDTA; 5% CO_2_; 37^Ŷ^C.
- Culture Medium: EMEM (EBSS) + 2mM Glutamine + 1% Non Essential Amino Acids (NEAA) + 1mM Sodium Pyruvate (NaP) + 10% Foetal Bovine Serum (FBS).
- Karyotype: 2n = 46
- Markers: Positive for GFAP
- Receptors: Over expression of EGFR

**Fig. 1:**
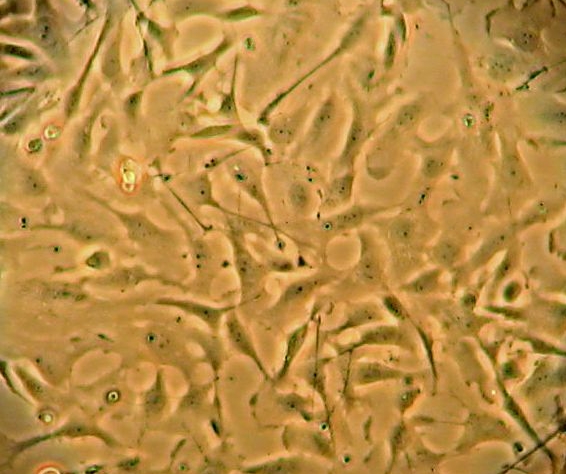
Microscopical aspects of Glioblastoma line U 87(Inverted microscope, X20)

### Green fluorescence protein gene (GFAP) transfection 

Transfection method involved lipofectamin, according to the manufacturer's protocol (Invitrogen). The type of vector is shuttle: it can replicate in prokaryote and in eukaryote also. Cells were mixed together with plasmidic DNA (vector with GFAP gene) and lipofectamine in 6 wells–one million cells/well. Then they were kept for 24 h at 37^Ŷ^C. The GFAP expression was evaluate at fluorescence microscopy

**Fig. 2:**
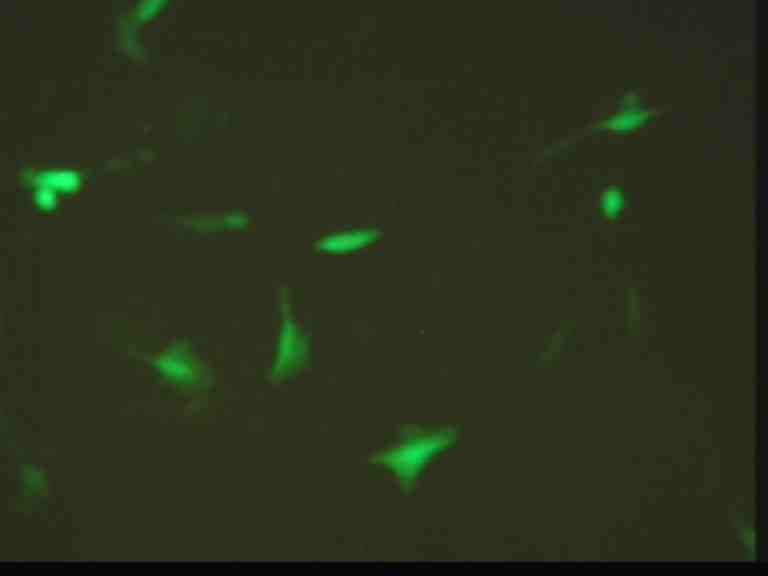
U 87 cells transfected with GFP–visualized at fluorescence microscope

### Nude Mice

The athymic mice (nude mice) were acquired from the Charles River laboratories Germany. The full name of this line is: NUDE Crl: CD–1 Foxn1 and was obtained by successive transfers of the nude gene into mice CD–1. These mice are athymic and have albinos, hairless phenotype.

**Fig. 3:**
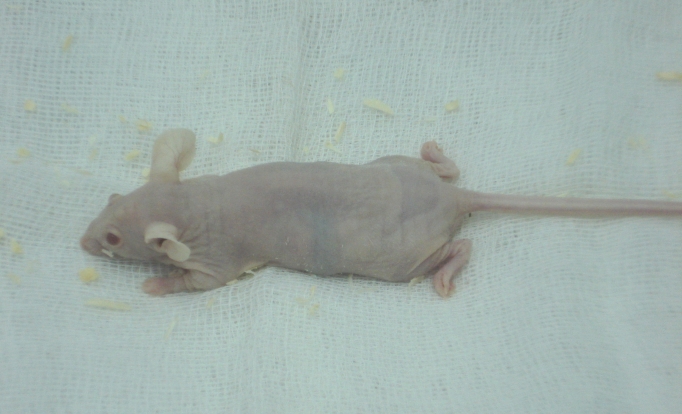
The phenotype of the nude mice used in our experiments

Comparing with the other line of the Charles River laboratories, this line has the following characteristics:

**Table 1:** 

Line/characteristic	Hair	Thymus	T–cells	B–cells	NK cells	Fix complement
**CD1 nude**	–	–	–	+	+	+
Nu/Nu nude	–	–	–	+	+	+
Balbc/nude	–	–	–	+	+	+
NIH–3 nude	–	–	–	–	+	+
Fox Chase SCID	+	+	–	–	+	+

### Instruments

For the scalp incision we used a set of micro–surgical forceps, scissors and grips (Aesculap, Germany). 

**Fig. 4:**
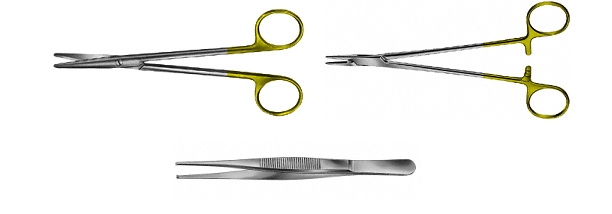
Micro–instruments used for surgical experiments

In order to make the burr whole in the skull as small as possible, we used a special electric drill system (TC–Motor 3000, Nouvag, Switzerland). This system has an electronic control of the speed and uses small drills (1mm diameter).

**Fig. 5:**
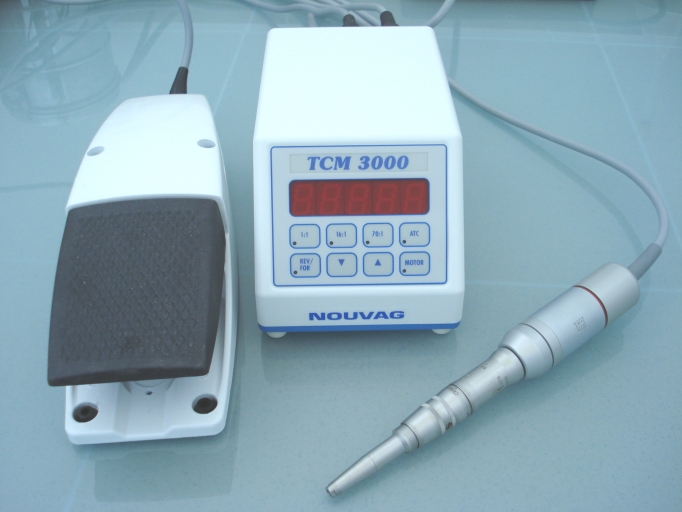
Micro–motor TC–Motor 3000

The inoculation of the glioblastoma cells have been performed stereotactically with a special stereotactic system (TAXIC–600 #x2013; WPI Stereotaxic Frame 18 deg. Ear Bars and UMP 3–1 injection system), (World Precision Instruments, Germany). This system enables researchers to inoculate very small volumes of glioblastoma cells suspension (3 micro liters) during a controlled period of time (3 minutes) very precisely, targeting the same structures of the mice brain.

**Fig. 6:**
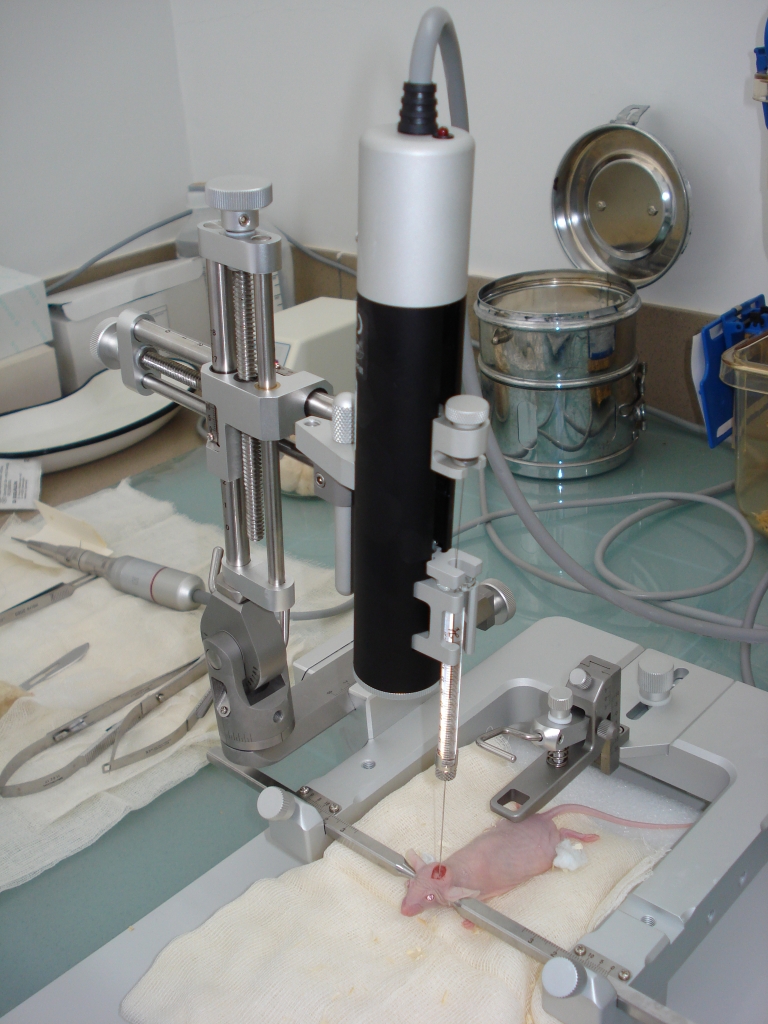
Stereotaxic system: TAXIC–600 – WPI Stereotaxic Frame 18 deg. Ear Bars and UMP 3–1 injection system, (World Precision Instruments, Germany).

## Meth

### Anesthetic technique

After we had tried several anesthetic techniques described in the literature, we selected the most efficient and easy to administered one. This is a combination of ketamine (between 80 and 100mg/kg) and xylazine (10mg/kg). These drugs are combined in the same syringe and are administered intraperitoneally. The anesthetic effect is obtained within 5 minute after the drugs administration. The effect lasts between 20 and 30 minutes, and produces a good muscular relaxation and doesn't required any specific parameters controls (respiration, cardiac monitoring).

**Table 2:** 

Agent	Doze	Anesthetic period
Pentobarbital>	50 mg/kg IP	20–40 minutes
Tribromoethanol (avertin)	240 mg/kg IP	15–45 minutes
Metomidate/fentanyl	60 mg/kg + 0.06 mg/kg SC	20–30 minutes
**Ketamine/xylazine**	**80–100 mg/kg + 10 mg/kg IP **	20–30 minutes

### Inoculation technique

We used as anatomical landmark bregma, which is easy to be recognized after skin incision

The burr whole is performed 3mm lateral and 1mm in front of the bregma, using a small drill (1mm diameter).

**Fig. 7:**
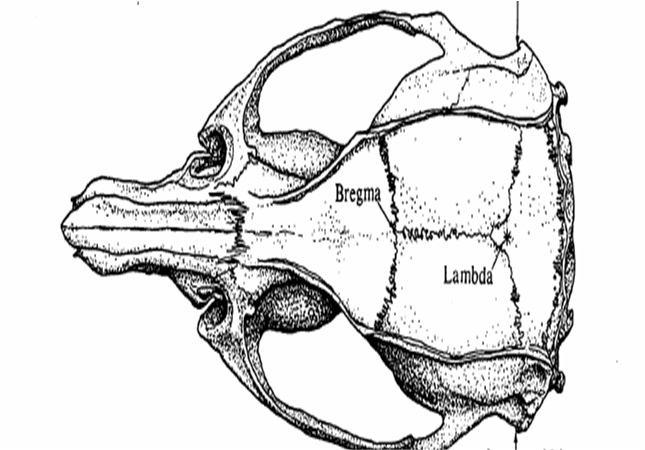
The main anatomical landmarks

Then we introduce stereotactically the needle of the Hamilton syringe until a depth of 3,5mm from dura mater, with a 30 grades tilt of the needle in the coronal plane from laterally to medially. 

**Fig. 8:**
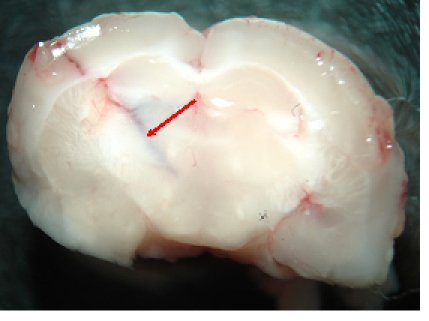
Inoculation of trypane blue in order to demonstrate the site of inoculation–coronal section of the mouse brain

The next step is to inoculate, within 3min, approximately 500.000 glioblastoma cells, suspended in 3microL, using the electronic control of the injection system. The needle is left in place for one more minute after finishing the cells inoculation then is removed slowly. We applied sterile wax inside the bur whole then sutured the scalp. 

**Fig. 9:**
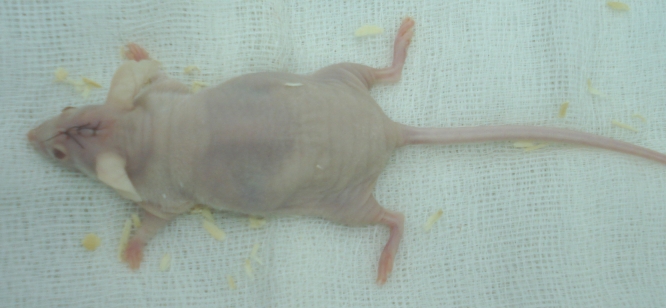
Mouse post–inoculation–aspect of scalp incision

We established two lots:

- Lot 1 (20 mice). For this lot we inoculated 5×10^5^ U87 native glioblastoma cells
- Lot 2 (10 mice). In this lot we inoculated 5×10^5^ U87 glioblastoma cells transfected with GFP gene.

The samples (mice brains) were obtained for both lots at specific intervals post-inoculation: 7 days, 14 days, 21 days and 28 days. Two mice, one for each lot, die at 24 post–inoculation. One mice of the lot 1 died spontaneously at 27 days. When the skull was opened the tumor developed and occupied more then a half of the hemisphere; this was probably the cause of mouse death.

All the animal manipulations have been performed in accordance with national and international guidelines. 

The samples have been sent to the Department of Histology of the University Bucharest in order to be performed the histology and immunohistochemistry studies.

### Histology

For morphologic analysis, mice brains were fixed in 4% formaldehyde in PBS, embedded in paraffin and 5–micrometer coronal sections were prepared. Sections were also stained with DAPI (4,6–diamidino–2–phenylindole) (1milig/mL) to visualize the nuclei. Fluorescent signals by either GFP or DAPI were examined under the fluorescence microscope (Zeiss Axiostar Plus).

### The Immunohistochemistry studies

8milim–thick sections were sequentially incubated in 3% H_2_O_2_ to remove endogenous peroxidase (10 min), washed PBS and incubated with 2% bovine serum albumin (BSA, fraction V) to remove non–specific background staining (30 min). Sections were incubated overnight, at 40 C with rabbit anti human b2 microglobulin peroxidase conjugated (Biomol), diluted 1:50, rinsed with PBS and incubated for 10–15 min in a solution of 0.05– 3,3–diaminobenzidine tetrachloride (DAB) and 0.015% hydrogen peroxide, dissolved in PBS. The nuclei were counterstained with haematoxylin (blue).

Coverslips with glioblastoma cells were rinsed four times with cold PBS and fixed in 4% formaldehyde in PBS. Fixed cells were washed in PBS and incubated with 2% BSA to remove non–specific background staining (30 min). The cells were subsequently incubated overnight at 4 degrees C with mouse monoclonal anti Glial Fibrillary Acidic Protein (GFAP), primary antibody (Sigma), diluted 1:150, followed by rinses in PBS and incubation with a secondary HRP–conjugated rabbit anti–mouse antibody (Rockland), diluted 1:400 (1h, at room temperature).

*The photomicrographs* were taken by digital camera (AxioCam MRc 5, Carl Zeiss) driven by software AxioVision 4.6 (Carl Zeiss). 

## Results

**Fig. 10:**
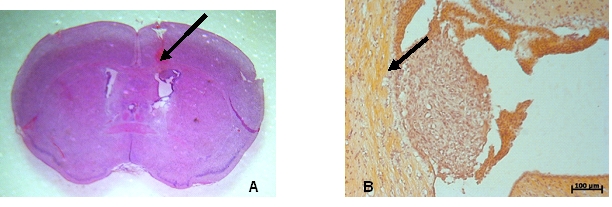
Macroscopic (10A) and microscopic (10 B–inverted image) aspect of the glioblastoma xenograft at 7 days postinoculation (arrow). Colored with hematoxilin–eosin.

The macroscopic and microscopic images performed at 7 days after glioblastoma inoculation, have shown the tumor developed at the injection site, near the wall of the lateral ventricle in the right hemisphere. (Fig. 10). 

**Fig. 11:**
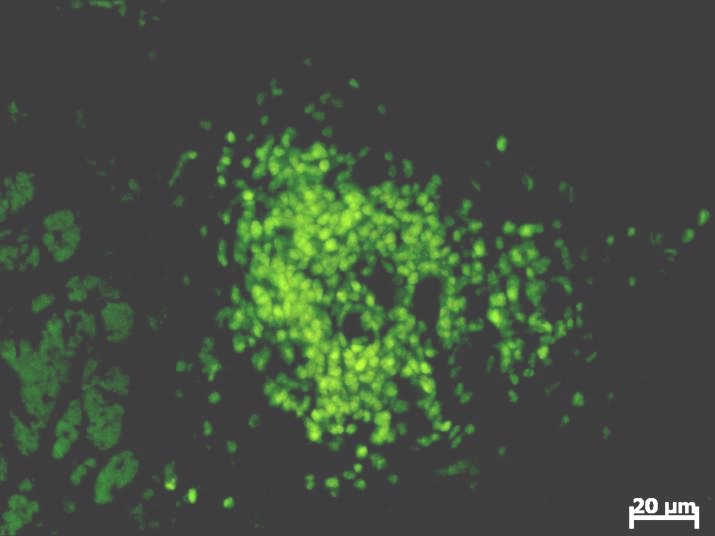
The fluorescence microscopic aspect of the GFP transfected glioblastoma cells at 3 days post–inoculation. Computerized system acquisition has been used for this image

**Fig. 12:**
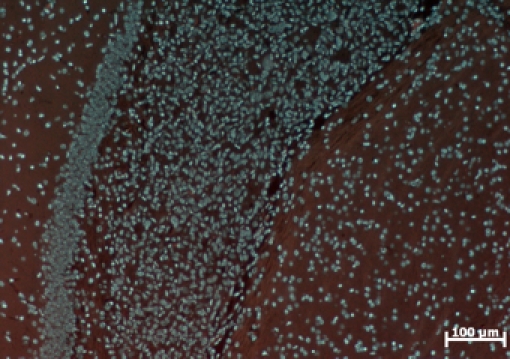
Tumor characterized by hyper–cellurality. Image taken at 7 days post inoculation (Colored with DAPI).

**Fig. 13:**
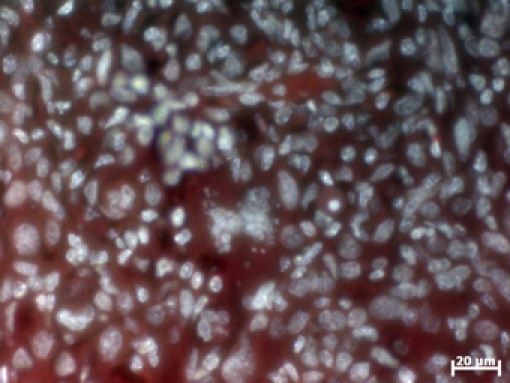
Multinucleate gigantic cells (arrow) Image taken at 7 days post inoculation (Colored with DAPI).

In fluorescence microscopy, the tumor is characterized by the presence of the following characteristics: hypercellularity, nuclear atypical aspect, aberrant mitotic figures and gigantic multinucleate cells (Fig. 11,Fig. 12, Fig. 13).

**Fig. 14:**
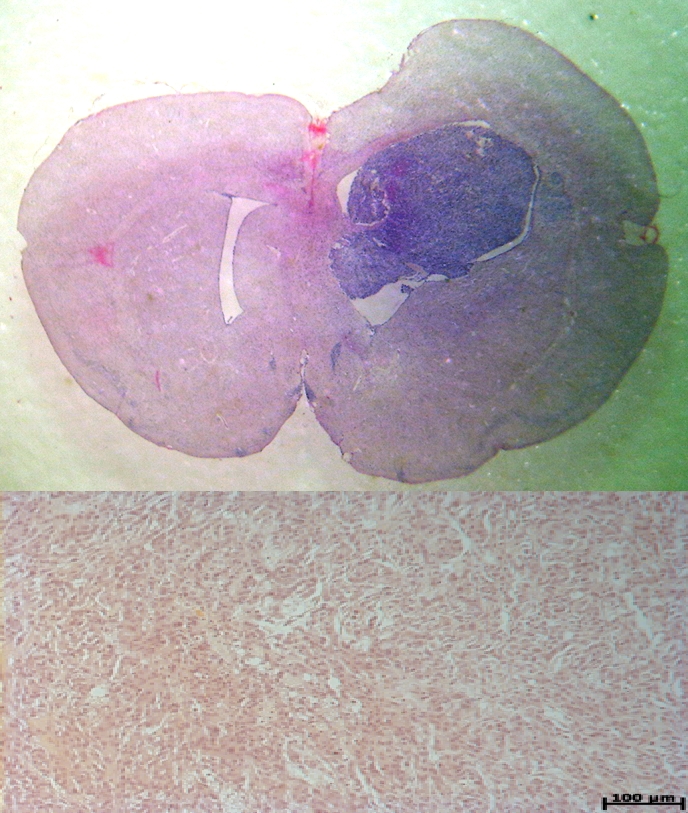
The glioblastoma induced tumor at 28 days post–inoculation (14 A). The microscopic aspect (14 B). Colored with hematoxilin–eozina.

At 28 days post inoculation, the tumor is very large, occupying almost a half of the right cerebral hemisphere (Fig. 14).

## Discussions

The poor prognosis of glioblastoma orientated the researcher to the direction of developing new molecular and cellular therapies. The first step in discovering new therapeutically agents is to test these on in vitro model of standardized glioblastoma cells cultures. The authors of this paper have succeeded for the first time in Romanian to develop a new glioblastoma cells line–T11 (more then 150 passages, characterized glioblastoma line) [1].

When an agent proved its efficacy on culture cells, the next step is to test this substance on animal model. Therefore, the development of an in vivo model is absolutely necessary, as an intermediate stage between in vitro experiments and the first phase of a clinical trial.

In order to develop the tumor in mice, using a human glioblastoma line, like U87MG, the animal should have the immune system ‘switched–of’. Thus, all the mice used for developing the model of glioblastoma are knock–out mice for a special gene–the FOX 1 gene. These mice are athymic mice and have severe deficiency in the production of T cells. The immunodeficiency of the mouse allows the inoculated human glioblastoma cells to develop the glioblastoma xenograft inside their body. The therapeutic response of various agents to brain tumor cell lines is frequently tested in immunodeficient mice following subcutaneous or intracerebral injection. The subcutaneous injection allows one to evaluate the response to treatment by simply sacrificing the animal at specific time intervals and measuring the size of the tumor, a technique that requires the use of a large number of animals and adds to the cost. However, a more frequently used method is to inject the mice intracerebral and to asses the volume of the developed tumor by scarified the animals at specific period of time.

The experimental intracranial injection techniques previously reported [2–12] do not necessarily or accurately assess the following parameters: the number of cells injected, their viability after injection, the degree of anatomical disruption of the injection site, whether the tumor cells are confined to the parenchyma or have been injected into the ventricles. Yamada et al (2004) (13) described in details several techniques aspects of injecting the gliobastoma cells in mice brain. First they define very precisely the target of the injection, then they assess the volume of glioblastoma cells, which should nod exceed the 3miliL, and the time of injection, not less then 3 minutes. Following these useful data, found in the literature, the authors established the protocol of inoculation before starting the experiments. Therefore, the basal ganglia was defined as the target area, using the landmarks previously described then 3miliL of glioblastoma cells suspension has been injected within 3 minutes. Using this method the mortality of the mice included in this study was low (10%) and the rate of success in developing the glioblastoma xenograft was high (90%).

Interestingly, Castro et al (2007) recently suggested that the number of U87cells required for developing the glioblastoma in nude mice is about 1 million cells [14]. However, in the experiments described above, authors succeeded to develop the glioblastoma using only 500.000 cells/inoculation, even they used the same materials and methods as Castro et al.

The characteristic of glioblastoma xenograft developed by authors were close to those described previously in literature (5, 7, 14). The authors performed classic microscopic studies, immunohistochemistry studies and microscopic fluorescence studies. The quality of images taken by a performing microscope and a digital acquisition system proved the success of the model, and ensure the reproducibility of the experiments.

## Conclusions

The authors of this paper managed to develop for the first time in Romania glioblastoma xenograft in nude mice. The experiments were reproducible with a high rate of success. Establishing an *in vivo* model for gliobastoma, authors proved that the collaboration between clinical hospitals and researcher institute is very important for developing new experimental models. Therefore, this achievement could be the start point for developing new projects and new experiments in order to find innovative therapies for glioblastoma. Testing newly discovered therapeutically agents on in vivo models will offer the researchers more secure in using these substances on human in clinical trials, and could be a link between fundamental researcher and clinical research.
